# The effect of spinal manipulative therapy on experimentally induced pain: a systematic literature review

**DOI:** 10.1186/2045-709X-20-26

**Published:** 2012-08-10

**Authors:** Mario Millan, Charlotte Leboeuf-Yde, Brian Budgell, Michel-Ange Amorim

**Affiliations:** 1 EA 4532 CIAMS, UFR STAPS, University Paris-Sud, Paris, France; 2 The Research Department, The Spine Centre of Southern Denmark Hospital Lillebælt, Lillebælt, Denmark; 3 Institut Franco-Européen de Chiropratique, Paris, France; 4 Institute of Regional Health Services Research, Faculty of Health Sciences, University of Southern Denmark, Odense, Denmark; 5 Canadian Memorial Chiropractic College, Toronto, ON, Canada; 6 Institut Universitaire de France, Paris, France

## Abstract

**Background:**

Although there is evidence that spinal manipulative therapy (SMT) can reduce pain, the mechanisms involved are not well established. There is a need to review the scientific literature to establish the evidence-base for the reduction of pain following SMT.

**Objectives:**

To determine if SMT can reduce experimentally induced pain, and if so, if the effect is i) only at the level of the treated spinal segment, ii) broader but in the same general region as SMT is performed, or iii) systemic.

**Design:**

A systematic critical literature review.

**Methods:**

A systematic search was performed for experimental studies on healthy volunteers and people without chronic syndromes, in which the immediate effect of SMT was tested. Articles selected were reviewed blindly by two authors. A summary quality score was calculated to indicate level of manuscript quality. Outcome was considered positive if the pain-reducing effect was statistically significant. Separate evidence tables were constructed with information relevant to each research question. Results were interpreted taking into account their manuscript quality.

**Results:**

Twenty-two articles were included, describing 43 experiments, primarily on pain produced by pressure (n = 27) or temperature (n = 9). Their quality was generally moderate. A hypoalgesic effect was shown in 19/27 experiments on pressure pain, produced by pressure in 3/9 on pain produced by temperature and in 6/7 tests on pain induced by other measures. Second pain provoked by temperature seems to respond to SMT but not first pain. Most studies revealed a local or regional hypoalgesic effect whereas a systematic effect was unclear. Manipulation of a “restricted motion segment” (“manipulable lesion”) seemed not to be essential to analgesia. In relation to outcome, there was no discernible difference between studies with higher vs. lower quality scores.

**Conclusions:**

These results indicate that SMT has a direct local/regional hypoalgesic effect on experimental pain for some types of stimuli. Further research is needed to determine i) if there is also a systemic effect, ii) the exact mechanisms by which SMT attenuates pain, and iii) whether this response is clinically significant.

## Background

### Pain

Pain is defined as an unpleasant sensory and emotional experience associated with actual or potential tissue damage [1]. It originates in specific receptors, named nociceptors, which are classified according to the type of damage to which they respond; thus, mechanosensitive, thermosensitive, chemosensitive and polymodal nociceptors.

From the peripheral nociceptors, noxious stimuli are transmitted to the dorsal horn of the spinal cord [2]. There, afferent fibers synapse in the superficial laminae of the dorsal gray matter of the spinal cord [3]. Cells in the superficial laminae serve as an integration centre and relay system for many sensations. Most cells of the grey matter involved in nociception send axons across the midline of the spinal cord to ascend to the thalamus. From there, they project upwards, eventually to the cortex of the brain. However, impulses are not only transmitted to the cerebral cortex. There are also other pathways and mechanisms that may participate in analgesic influences at the spinal and supraspinal levels.

When a noxious stimulus occurs, there may be a “first pain”, conducted by Aδ fibers, and a “second pain”, due to temporal sensory summation (TSS) and conducted by nociceptive C-fibers. “First pain” is described as sharp and "pricking". The propagation of this stimulus is relatively quick and it is felt in a well-defined part of the body surface [2]. “Second pain”, which is transmitted more slowly, is often described as dull and aching, and it is poorly localized. This pain tends to last beyond the termination of an acute noxious stimulus. Sources, pathways, perception of and treatment of the two types of pain are very different [4].

### Modulating pain

The infliction of pain is not always experienced in a linear manner according to the strength and nature of the stimulus. Pain sensations can be very different from one individual to another, and also intra-individual variations can occur, so that identical types of damage do not necessarily result in an identical amount and type of pain. One reason for this is that pain can be modulated, both to increase and decrease.

One modulating system, central sensitization, tends to increase pain sensation [5], particularly in people who have more long-lasting pain, making them more sensitive to “new” pain impulses than they would have been under normal circumstances.

Another endogenous modulating system is afferent or segmental inhibition, meaning that one external stimulus can block an ongoing pain sensation by having higher priority in reaching the brain [4].

Descending antinociceptive systems provide yet another modulating mechanism. These originate largely in the mesencephalon and have synaptic connections with neurons in the medulla and the spinal cord. This means that nociceptive information may be blocked or attenuated before it reaches higher centers [6]. This system is also tightly connected to a descending pain facilitating pathway that has the same general sites of origin (mesencephalon and medulla) but with the opposite effect.

Finally, there are also other intrinsic mechanisms for physiological modulation of pain, such as subjective assessment and motivational-affective modulation [7], which act by raising pain thresholds via endogenous opioids and other substances. These mechanisms, at times, preferentially alter sensory and/or affective aspects of pain perception, and the associated modulation of pain-evoked neural activity occurs in limbic and/or sensory brain regions, suggesting multiple endogenous pain-modulating systems [8]. Thus, pain can be increased or decreased by mere expectations or, even, abolished by feelings of, for example, fear.

### Treating back pain with spinal manipulative therapy

Although back pain is common and frequently distressing, and many therapies have arisen to treat it, musculoskeletal pain remains difficult to diagnose and treat. Spinal manipulative therapy (SMT) is one common treatment for musculoskeletal pain. One class of SMT involves a high-velocity, low-amplitude (HVLA) manipulation frequently used by chiropractors. HVLA treatments are mechanical events. They cause slight momentary deformations of the spine and surrounding soft tissues, and often elicit a cracking sound thought to be brought about by cavitation of spinal facet joints [9-11]. It is common to differentiate manipulation from mobilization. In the latter case, the joint is not taken beyond its passive limit. Rather mobilization can be described as a passive and perhaps repetitive stretch. Manipulation, on the other hand, carries the vertebrae beyond the normal physiological range of movement without exceeding the boundaries of anatomic integrity [11]. However, the distinction between manipulation and mobilization is probably not that clear, and it has been shown that cavitation is not necessary for SMT to exert a clinical effect [12-16]. The term SMT can therefore be used to describe various types of manual therapy (MT).

### The possible mechanisms of spinal manipulative therapy in back pain

Clinical experience indicates that both HVLA and mobilization, and also other types of manual therapy (MT), can have an immediate effect on pain. The literature also suggests that SMT has a direct neurological pain-reducing effect, by evoking one or possibly several of the physiologic pain-modulating mechanisms described briefly above. Indeed, there could be a combination of mechanisms or a number of these acting on different causes of pain. In this article we shall concentrate on the possible direct effect of SMT on pain. There are three possible levels of this hypothesized direct effect of SMT on pain, i.e. local, regional or central.

#### Local pain reducing effect

One theory is that SMT would have a pain-reducing effect primarily at the level of the manipulation, i.e. at a specific spinal level. In all, this phenomenon would probably be the result of a mix of different mechanisms. For example, SMT may mechanistically act to decrease the sensitivity of the muscle spindles and/or the various segmental sites of a reflex pathway [17].

#### Regional pain reducing effect

Another possibility is that SMT could have a regional effect, although still at the spinal level of the manipulative input. Some authors suggest an effect on the dorsal horn of the spinal cord [18] or on the periaqueductal grey area [19-21]. SMT is also thought to affect reflex neural outputs to both muscle and visceral organs by affecting both paraspinal muscle reflexes and motoneuron excitability [22].

#### Central pain reducing effect

Recently, it has been hypothesized that SMT reduces the potential for central sensitization by inhibiting TSS (“second pain”) [23]. One mechanism underlying the effects of SMT may be the ability of manipulation to alter central sensory processing by removing subthreshold mechanical or chemical stimuli from paraspinal tissues [22].

### Conflicting literature

Also a comprehensive model of mechanisms of manual therapy in the treatment of musculoskeletal pain has been suggested [24] consisting of a cascade of neurophysiological responses from the peripheral and central nervous systems which are then responsible for the clinical outcomes. In other words, the literature offers many possible mechanisms and combinations of mechanisms to explain the pain-reducing effect of SMT. In fact, there is a lot of information available in the literature on this topic. However, the literature is difficult to grasp and conflicting because it consists of a mixture of discussions, hypotheses, and studies employing different designs, methods and outcome variables. Therefore, there is a need for systematic and critical literature reviews in order to establish the evidence-base for various theories relating to the direct or indirect reduction of pain following SMT. A first step might be to establish the weight of evidence in relation to whether pain is indeed dampened by the application of SMT to the spinal structures.

### Aims and objectives

Therefore, in this systematic critical literature review we shall examine the effect of spinal manipulation on experimentally induced pain in healthy study subjects concentrating on the possible direct effects of SMT on pain at three levels, i.e. local, regional or systemic. Because different types of pain may travel through different pathways, these were studied separately.

The specific research questions were:

1- Does SMT reduce pain provoked by pressure?

2- Does SMT reduce pain provoked by temperature?

3- Does SMT reduce pain provoked by methods other than pressure and temperature?

4- Does SMT reduce experimentally induced pain at the spinal segment where it is performed?

5- Does SMT reduce experimentally induced pain in the spinal region where it is performed?

6- Does SMT have a systemic (global) effect on experimentally induced pain?

## Methods

In order to obtain answers to the questions above, we undertook a systematic critical literature review, which commenced with a systematic literature search of PubMed, Mantis, and the Cochrane Library using specific search terms. These search terms were: Spinal manipulation pain: _("manipulation, spinal"[MeSH Terms] OR ("manipulation"[All Fields] AND "spinal"[All Fields]) OR "spinal manipulation"[All Fields] OR ("spinal"[All Fields] AND "manipulation"[All Fields])) AND ("pain"[MeSH Terms] OR "pain"[All Fields]) ;_ Chiropractic manipulation pain**:**_("manipulation, chiropractic"[MeSH Terms] OR ("manipulation"[All Fields] AND "chiropractic"[All Fields]) OR "chiropractic manipulation"[All Fields] OR ("chiropractic"[All Fields] AND "manipulation"[All Fields])) AND ("pain"[MeSH Terms] OR "pain"[All Fields]) ;_ Spinal manipulation experimental pain: _("manipulation, spinal"[MeSH Terms] OR ("manipulation"[All Fields] AND "spinal"[All Fields]) OR "spinal manipulation"[All Fields] OR ("spinal"[All Fields] AND "manipulation"[All Fields])) AND experimental[All Fields] AND ("pain"[MeSH Terms] OR "pain"[All Fields]) ;_ and Chiropractic manipulation experimental pain: _("manipulation, chiropractic"[MeSH Terms] OR ("manipulation"[All Fields] AND "chiropractic"[All Fields]) OR "chiropractic manipulation"[All Fields] OR ("chiropractic"[All Fields] AND "manipulation"[All Fields])) AND experimental[All Fields] AND ("pain"[MeSH Terms] OR "pain"[All Fields])._

Inclusion and exclusion criteria (see Additional file 1) were applied by the first author to the titles and abstracts of the studies identified in the search. Once most selected articles were retrieved, a citation search was made based on the retrieved articles’ reference lists. All articles selected were reviewed separately by two different authors blinded to each other’s evaluations. Each author separately extracted data from every article according to a checklist. Data were later compared in order to minimize extraction errors. The fourth author would arbitrate any disagreement between the two reviewers. An ongoing search was performed until December 31, 2011 and the review process was repeated when new articles were found.

A table was constructed in order to describe the selected articles, as shown in Table 1.

**Table 1:** Descriptive items used in a systematic critical literature review on the effect of SMT on pain

**Reference**	**Year**	**Authors**	**Title**	**Location**	**Setting**	**n° of subjects**	**n° males**	**n° females**	**Ages**	**Treatment groups**	**How was pain produced**	**How was pain measured**	**When was pain measured**	**Description of study subjects**	**Approval ethics committee**
[37]	2011	Josue Fernández-Carnero, Joshua A. Cleland and Roy La Touche Arbizu	Examination of motor and hypoalgesic effects of cervical vs thoracic spine manipulation in patients with lateral epicondyalgia: a clinical trial.	Spain	University	18?	8	9	44.8 SD, 9.2 (30–60)	- Cervical manipulation	Pressure	Electronic digital algometer	Before and after	Faculty of the Health Science	Yes
										- Thoracic manipulation					
[42]	2011	V. Maduro de Camargo, F. Alburquerque-Sendín, F. Bérzin, Vinicius Cobos Stefanelli, D. P. Rodrigues de Souza and C. Fernández-de-las-Peñas,	Immediate effects on electromyographic activity and pressure pain thresholds after a cervical manipulation in mechanical neck pain: a randomized controlled trial.	Brazil	University	37	21	16	18 – 42	- SMT C5-C6	Pressure	Analogue algometer	Before and after	University workers	Yes
										- Control					
[23]	2011	Mark D. Bishop, Jason M. Beneciuk, Steven Z. George;	Immediate reduction in temporal sensory summation after thoracic spinal manipulation.	USA	University	90	24	66	22.9 + −2.7	- SMT	T° and pressure	Algometer	Before and Immediately after	Students	No
										- Cervical exercises					
										- Control					
[28]	2010	Benjamin Soon, Annina B. Schmid, Elias J. Fridriksson, Elizabeth Gresslos, Philip Cheong and Anthony Wright;	A crossover study on the effect of cervical mobilization on motor function and pressure pain threshold in pain-free individuals.	Australia	University	24	13	11	34 +/−12	- Mobilization	Pressure	Digital algometer	Before and after	Students	Yes
										- Manual contact control					
										- Control					
[29]	2010	Oliveira-Campelo NM, Rubens-Rebelatto J, Martín-Vallejo FJ, Alburquerque-Sendí N F, Fernández-de-Las-Peñas C.	The immediate effects of atlanto-occipital joint manipulation and suboccipital muscle inhibition technique on active mouth opening and pressure pain sensitivity over latent myofascial trigger points in the masticatory muscles.	Spain	Osteopathic school and university	122	31	91	18-30	- Manipulation	Pressure	Mechanical algometer	Before and 2 min post treatment	Students	Yes
				Brazil						- Soft tissue					
										- Control					
[44]	2010	Elaine Willett, Clair Hebron and Oliver Krouwel	The initial effects of different rates of lumbar mobilizations on pressure pain thresholds in asymptomatic subjects.	UK	University	30	8	22	33.05 (18–57)	- 2 Hz	Pressure	Electronic algometer	Base	11 naive physiotherapists 19 non naive	Yes
										- 1 Hz			+ 48 h		
										- Quasi stable			+ 48 h		
[38]	2009	P. Mansilla-Ferragut, C. Fernández-de-las Peñas, F Alburquerque-Sendin, J. A. Cleland and JJ Boscá-Gandia	Immediate effects of atlanto-occipital joint	Spain	Osteopathic school	37	0	37	35 +/−8	- SMT	Pressure	Mechanical algometer	Before and after	Volunteers, general population	Yes
			Manipulation on active mouth opening and pressure pain sensitivity in women with mechanical neck pain.												
										- Control					
[30]	2009	Oliver Thomson, Lesley Haig, Hazel Mansfield	The effects of high-velocity low-amplitude thrust manipulation and mobilization techniques on pressure pain threshold in the lumbar spine.	Sweden	Stockholm College Osteopathic	50	29	21	27	- unilateral HVLAT	Pressure	Pressure algometer	Before and after	Students	Yes
				UK	School British College					- Spinal lumbar mobilization					
										- Sham laser procedure					
[43]	2009	Oliver Krouwel , Clair Hebron, Elaine Willett	An investigation into the potential hypoalgesic effects of different amplitudes of PA mobilizations on the lumbar spine as measured by pressure pain thresholds.	UK	University	30	9	21	26,43 (SD 4,92)	- Large oscillation (force applied)	Pressure	Digital algometer	Baseline before and + 24, + 24	13/30 physiotherapy naives	Yes
										- Small oscillation quasi static					
[18]	2009	Joel E. Bialosky, Mark D. Bishop, Michael E. Robinson, Giorgio Zeppieri Jr, Steven Z. George	Spinal manipulative therapy has an immediate effect on thermal pain sensitivity in people with low back pain: a randomized controlled trial.	USA	University	36	10	26	32.38 (12.63)	- SMT	T°	Numerical scale	Before and Immediately after	Students with low back pain	No
										- Biking					
										- Back extension exercise					
[19]	2008	J. Fernández-Carnero, Cesar Fernández-de-las-Peñas, and Joshua A. Cleland	Immediate hypoalgesic and motor effects after a single cervical manipulation in subjects with lateral epicondyalgia.	Spain	Universities and Osteopathic school Madrid	10	5	5	42 (SD6)	- Manipulative session	T° and pressure	Electronic algometer	Before and after	Patients	Yes
				USA						- Manual contact intervention					
[39]	2008	C. Fernández-de-las-Peñas, C. Alonso-Blanco, J. A. Cleland, C. Rodriguez-Blanco and F.Alburquerque-Sendin	Changes in pressure pain thresholds over C5-C6 zygapophyseal joint after a cervicothoracic junction manipulation in healthy subjects.	Spain	Universities and Osteopathic school Madrid	30	13	17	26 (SD 5)	- Manipulative thrust right side C7-T1	Pressure	Algometer	Before and after	General population	Yes
										- Manipulative thrust left side C7-T1					
				USA						- Sham-manual procedure					
[20]	2007	M. Ruiz-Sáez, C. Fernández-de-las-Peñas, C. Rodriguez Blanco, R. Martinez-Segura and R. Garcia-León	Changes in pressure pain sensitivity in latent myofascial trigger points in the upper trapezius muscle after a cervical spine manipulation in pain-free subjects.	Spain	Osteopathic school	72	27	46	31 (SD10)	- Manipulative	Pressure	Mechanical algometer	Baseline before 1, 5 and 10 min after	Volunteers, general population	Yes
										- Sham-manual					
[31]	2007	Fernández-de-las-Peñas C, Pérez-de-Heredia M, Brea-Rivero M, Miangolarra-Page JC.	Immediate effects on pressure pain threshold following a single cervical spine manipulation in healthy subjects.	Spain	Universities	15	7	8	21 + −2	- Manipulation	Pressure	Mechanical algometer	Before 5 min after intervention	Students	Yes
										- Placebo			3 sessions separated by 48 h		
										- Control					
[34]	2007	Hamilton L, Boswell C, Fryer G	The effects of high-velocity, low-amplitude manipulation and muscle energy technique on suboccipital tenderness.	Australia	University	90	29	61	23 +/−5	- SMT (C0- C1)	Pressure	Electronic algometer	Before and after	Students	Yes
										- Muscle energy technique					
										- Control					
[36]	2006	George SZ, Bishop MD, Bialosky JE, Zeppieri G Jr, Robinson ME.	Immediate effects of spinal manipulation on thermal pain sensitivity: an experimental study.	USA	University	60	20	40	24.03(SD 3.2°	- SMT	T°	Medoc neurosensory analyzer	Before and 5 min after	Students	Yes
										- Lumbar ext exercise					
										- Bicycle riding					
[40]	2004	P.Mohammadi, A. Gonsalves, Chris Tsai, T. Hummel and Thomas Carpenter	Areas of capsaicin-induced secondary hyperalgesia and allodynia are reduced by a single chiropractic adjustment: preliminary study.	USA	Universities	20	14	6	27 (21–37)	- SMT	Cutaneous capsaicin	Visual Analogue Scale	Before and 20 min after after	Healthy volunteers, mostly naive to SMT	Yes
				Germany						- Non-SMT					
[35]	2004	Fryer G, Carub J, McIver S.	The effect of manipulation and mobilization on pressure pain thresholds in the thoracic spine.	Australia	University	96	39	57	19-34	- SMT T2-T4	Pressure	Electronic algometer	Before and after	Students	Yes
										- Mobilisation					
										- Control					
[21]	2001	M. Sterling, G. Jull, A. Wright	Cervical mobilization: concurrent effects on pain, sympathetic nervous system activity and motor activity.	Canada	University	30	14	16	35.7 (SD 14.92)	- SMT	Pressure / T°	Visual Analogue Scale, electronic algometer	Before and after	Patients pain +3 months C5/6	Yes
										- Placebo					
										- Control					
[32]	1998	Bill Vicenzino, David Collins and Anthony Wright	An investigation of the Interrelationship between manipulative therapy-Induced hypoalgesia and sympathoexcitation.	Australia	University	24	11	13	49.0 (27–70)	- Mobilization C5-C6	Pressure / T°	Visual Analogue Scale, digital algometer	Before and after	Patients epicond 6.2 +/− 5.1 months	Yes
										- Manual contact placebo					
										- Nothing					
[33]	1996	Bill Vicenzino, David Collins and Anthony Wright	The initial effects of a cervical spine manipulative physiotherapy treatment on the pain and dysfunction of lateral epicondyalgia.	Australia	University	15	7	8	44 +/−2	- Treatment	Pressure	Visual Analogue Scale digital algometer	Before and after	Patients epicond 8 +/− 2 months	Yes
										- Placebo					
										- Control					
[41]	1984	Terrett AC, Vernon H.	Manipulation and pain tolerance. A controlled study of the effect of spinal manipulation on paraspinal cutaneous pain tolerance levels.	Canada	Chiropractic college	50	?	?	28.6	- Thoracic manipulation	Electrical induction	Thresholds	Before and after	Chiropractic students	No
										- Control group					

T° = temperature.

Articles are presented consecutively by year of publication and identified by a number corresponding to its reference in the first column of each table. As we were unable to locate a suitable quality check-list for this type of research, a second set of criteria was developed in order to evaluate the quality and risk of bias in this type of research. We designed this checklist based on concepts presented in the PRISMA statement [25], the CONSORT statement [26] and Cochrane guidelines [27] bearing in mind that there can be no general recipe for such work, as review procedures have to be topic specific. The items selected for the quality checklist and their rationale have been described in Additional file 2. The risk of bias was assessed following the criteria suggested by the method guidelines for systematic reviews of trials of treatments for neck and back pain by Furlan et al [27] (see items 1, 2, 4, 6, 9, and 11 in Additional file 2). Additional items were mainly adapted from PRISMA [25] (see items 3, 5, 7, 8, 10, 12, and 13). Finally, the outcomes were noted for each experiment as a positive effect, negative effect, or no effect.

### Classifying articles by their quality

Each article selected was checked for each quality item. By consensus, we decided to give one point for every criterion, except for the 4 items that we considered as most important: a- unbiased/blind/naïve study subjects (item 1), b- random allocation (item 3), c- blinded assessment (item 9), and d- losses and exclusions (item 11). Each of these was assigned two points.

A summary quality score was calculated but no cutoff-point was defined for acceptable or unacceptable level of quality. This allowed us (and the readers) to use the quality scores and the information on each individual quality item as a guide for whether articles would be considered more or less credible. In other words, the quality assessment was meant to be flexible.

A post hoc comparison was made between results of the two quality scores (the total score and the score for the most important criteria). Using thresholds set at 12/18 and 4/8 points, respectively, the distributions of studies with positive and non-positive outcomes were compared for the two scales and in relation to whether the studies were of higher or lower quality.

### Data synthesis

The data tables were used, in a systematic fashion, to obtain answers to our research questions. This was done by highlighting relevant information to facilitate a visual representation (green = positive outcome, red = non-positive outcome) to make interpretation easier. Finally, studies were sorted in descending order starting with those with the highest total quality score out of 18. Results were thereafter interpreted and reported in a narrative fashion. If high and low quality studies generally obtained similar outcomes, we assumed that the poorer quality studies provided supporting evidence for the better studies. If, however, it was mainly the poorer quality studies which obtained positive findings, particularly if the assessors were not blinded or the study subjects could have been biased, then we would be more cautious in our interpretation of the results.

Because we decided not to use a scoring system to establish level of quality, we have not defined discrete levels of evidence.

## Results

In all, 1279 titles satisfying the inclusion criteria were identified in the initial PubMed search. Other database search results were as follows: a- Spinal manipulation pain: 1276 results in Pubmed, 10 in Mantis and 9 Cochrane Reviews. b- Chiropractic manipulation pain: 806 results in Pubmed, 2 in Mantis and 5 Cochrane Reviews. c- Spinal manipulation experimental pain: 63 results in Pubmed, 0 in Mantis and 13 Cochrane Reviews. d- Chiropractic manipulation experimental pain: 28 results in Pubmed, 0 in Mantis and 0 Cochrane Reviews. Only 5 articles were added after an additional search of reference lists.

All articles found in the Mantis and Cochrane databases were already identified via the Pubmed searches. Of the 1279 references, 116 were retrieved in full text for further scrutiny.

### Description of studies

Upon scrutinizing the full texts, 22 articles were found to fulfill the inclusion criteria. They described 43 experiments: 27 with pain produced by pressure, 9 by temperature, 3 by capsaicin, 2 examined spontaneous pain, 1 used a stretch test to produce pain and 1 used electrically induced pain. All were controlled trials published in English. Detailed information is presented in Table 1 and briefly summarized below.

Three research groups were responsible for 13 publications. They did not seem to have repeatedly used the same study samples but appeared to have reported on different study populations for the various studies. In most cases (n = 18), experiments were carried out totally or partially in universities. The number of subjects ranged from 10 to 122. Six articles used patients as study subjects; most of the others included non-clinical populations, often students. There were no studies with animals fulfilling our inclusion criteria.

Most of experiments (n = 20) used external control groups. Twelve of them used three groups: a treatment, a placebo and a control group in which no action was taken - noted in tables as “nothing” (studies [21,23,28-35]). Studies [18,36] used bicycle exercise as their control activity. Seven studies ( [19,20,37-41]) used a sham procedure as their control treatment, whereas study [42] compared the treatment group to a control group without a sham procedure. Two studies used only internal control groups; [43] and [44] compared three different types of mobilization.

### Data synthesis: Quality of studies

There were no disagreements between reviewers on the scoring of the manuscripts based on the checklists. Table 2 summarizes the quality items for each article. The quality and risk of bias assessment of studies reviewed revealed them to be relatively homogeneous, with the summary scores ranging between 8 and 16 out of 18 points, with mean and median summary scores of 12.2 and 13, respectively.

**Table 2:** Quality criteria of articles selected for a systematic critical literature review on the effect of SMT on pain

**Article**	**Is the assessment blinded?**		**Is there a control on psychological characteristics of subjects?**		**The validity of the outcome variable:**		**When was it measured?**		**Is the random procedure mentioned?**		**Number of experiments (pain + SMT)**		**Study subjects:**		**SMT performed by:**		**Is the SMT well described?**		**Are losses and exclusions reported?**		**Experimental conditions**		**Estimates given**		**Are differences tested for statistical significance?**		**Total of points**
	Yes = 2 pts No = 0 point	Points	Yes = 1 point No = 0 point	Points	Pilot study = 1 pts or ,Ref are given = 1 pt or, it's reproducible = 1 pt Nothing = 0 pt	Points (max 1 pt)	before and after = 1 point only after = 0 point	Points	Yes = 2 pts No = 0 point	Points	> 1 = 1 point 1 = 0 point	Points	Naive to tx and blind (sham manip) = 2 pts Naive or blind = 1 point Not naive and not blind = 0 pt	Points	Same person = 1 point Experienced person (> 5 years) = 1 point	Points (max 2 pts)	Yes = 1 point No = 0 point	Points	Yes = 2 pts No = 0 point	Points	same day or T° controlled or same time = 1 point different day and T° or time not controlled = 0 point	Points	Yes = 1 point No = 0 point	Points	Yes = 1 point No = 0 point	Points	Min = 0 Max = 18
[39]	Yes	2	No	0	Ref given	1	Before and after	1	Yes	2	3	1	Blind	1	Same and experienced	2	Yes	1	Yes	2	Same day	1	Yes	1	Yes	1	16
[21]	Yes	2	No	0	Reproducible	1	Before and after	1	Yes	2	3	1	Naive and blind	2	Same and experienced	2	Yes	1	No	0	Same day	1	Yes	1	Yes	1	15
[31]	Yes	2	No	0	Ref given	1	Before and after	1	Yes	2	3	1	Blind	1	Experienced	1	Yes	1	Yes	2	T controlled	1	Yes	1	Yes	1	15
[42]	Yes	2	No	0	Ref given	1	Before and after	1	Yes	2	3	1	Blind	1	Same and experienced	2	Yes	1	No	0	Same day	1	Yes	1	Yes	1	14
[29]	Yes	2	No	0	Ref given	1	Before and after	1	Yes	2	3	1	Naive	1	Same and experienced	2	Yes	1	No	0	Same day	1	Yes	1	Yes	1	14
[44]	No	0	No	0	Ref given	1	Before and after	1	Yes	2	3	1	Naive and blind	2	Same and experienced	2	Yes	1	Yes	2	More 48 H, no control T°	0	Yes	1	Yes	1	14
[38]	Yes	2	No	0	Ref given	1	Before and after	1	Yes	2	3	1	Blind	1	Same and experienced	2	Yes	1	No	0	Same day	1	Yes	1	Yes	1	14
[34]	Yes	2	No	0	Ref given	1	Before and after	1	Yes	2	3	1	Naive	1	Same and experienced	2	Yes	1	No	0	Same day?	1	Yes	1	Yes	1	14
[35]	Yes	2	No	0	Ref given	1	Before and after	1	Yes	2	3	1	Naive	1	Same and experienced	2	Yes	1	No	0	Same day?	1	Yes	1	Yes	1	14
[37]	Yes	2	No	0	Ref given	1	Before and after	1	Yes	2	3	1	Nothing	0	Same and experienced	2	Yes	1	No	0	Same day	1	Yes	1	Yes	1	13
[28]	Yes	2	No	0	Ref given	1	Before and after	1	Yes	2	3	1	Naive and blind	2	Experienced	1	Yes	1	No	0	More 48 H, no control T°	0	Yes	1	Yes	1	13
[43]	No	0	No	0	Ref given	1	Before and after	1	Yes	2	3	1	Naive and blind	2	Same	1	Yes	1	Yes	2	More 3 days, no control T°	0	Yes	1	Yes	1	13
[20]	Yes	2	No	0	Ref given	1	Before and after	1	Yes	2	3	1	Blind	1	Experienced	1	Yes	1	No	0	Same day	1	Yes	1	Yes	1	13
[40]	Yes	2	No	0	Reproducible	1	Before and after	1	Yes	2	2	1	Naive and blind	2	Experienced	1	Yes	1	No	0	More 7 days, no control T°	0	Yes	1	Yes	1	13
[18]	No	0	Yes	1	Ref given	1	Before and after	1	Yes	2	2	1	Nothing	0	Nothing	0	Yes	1	Yes	2	Same day	1	Yes	1	Yes	1	12
[19]	Yes	2	No	0	Ref given	1	Before and after	1	No	0	3	1	Blind	1	Same and experienced	2	Yes	1	No	0	More 48 H, no control T°	0	Yes	1	Yes	1	11
[32]	Yes	2	No	0	Ref given	1	Before and after	1	No	0	3	1	Naive and blind	2	Nothing	0	Yes	1	No	0	More 3 days, control T°	1	Yes	1	Yes	1	11
[30]	Yes	2	No	0	Ref given	1	Before and after	1	No	0	3	1	Blind to sham laser	1	Nothing	0	Yes	1	No	0	Same day	1	Yes	1	Yes	1	10
[36]	No	0	Yes	1	Ref given	1	Before and after	1	Yes	2	10	1	Nothing	0	Nothing	0	Yes	1	No	0	Same day	1	Yes	1	Yes	1	10
[41]	Yes	2	No	0	Ref given	1	Before and after	1	Yes	2	1	0	Nothing	0	Nothing	0	Yes	1	No	0	Same day	1	Yes	1	Yes	1	10
[33]	Yes	2	No	0	Ref given	1	Before and after	1	No	0	3	1	Naive and blind	2	Nothing	0	No	0	No	0	More 3 days, no control T°	0	Yes	1	Yes	1	9
[23]	No	0	Yes	1	Ref given	1	Before and after	1	No	0	2	1	Nothing	0	Nothing	0	yes	1	No	0	Highly controlled	1	Yes	1	Yes	1	8

T° = temperature.

**Table 2a:** A comparison between total scores obtained from two different scales (8 point scale and 18 point scale)

**Article**	**Score Max = 8**	**Score Max = 18**
[39]	6	16
[31]	7	15
[21]	6	15
[42]	5	14
[29]	5	14
[44]	6	14
[38]	5	14
[35]	5	14
[34]	5	14
[28]	6	13
[43]	6	13
[20]	5	13
[40]	6	13
[37]	4	13
[18]	4	12
[19]	3	11
[32]	4	11
[30]	3	10
[36]	2	10
[41]	4	10
[33]	4	9
[23]	0	8

Articles scoring < 4/8 or < 12/18 are shown in red.

**Table 2b:** Four main quality criteria of articles from literature review on effect of SMT on pain – maximum score 8 points

**Article**	**Is the assessment blinded?**		**Is the randomization procedure mentioned?**		**Study subjects:**		**Were losses and exclusions reported?**		**Total points**
	**Yes = 2 pts**	**Points**	**Yes = 2 pts**	**Points**	**Naive to tx and blind**	**Points**	**Yes = 2 pts**	**Points**	**Max = 8 Min = 0**
[31]	Yes	2	Yes	2	Blind	1	Yes	2	7
[28]	Yes	2	Yes	2	Naive and blind	2	No	0	6
[44]	No	0	Yes	2	Naive and blind	2	Yes	2	6
[43]	No	0	Yes	2	Naive and blind	2	Yes	2	6
[40]	Yes	2	Yes	2	Naive and blind	2	No	0	6
[21]	Yes	2	Yes	2	Naive and blind	2	No	0	6
[39]	Yes	2	Yes	2	Blind	1	Yes	2	6
[42]	Yes	2	Yes	2	Blind	1	No	0	5
[29]	Yes	2	Yes	2	Naive	1	No	0	5
[38]	Yes	2	Yes	2	Blind	1	No	0	5
[20]	Yes	2	Yes	2	Blind	1	No	0	5
[34]	Yes	2	Yes	2	Naive	1	No	0	5
[35]	Yes	2	Yes	2	Naive	1	No	0	5
[37]	Yes	2	Yes	2	Nothing	0	No	0	4
[18]	No	0	Yes	2	Nothing	0	Yes	2	4
[32]	Yes	2	No	0	Naive and blind	2	No	0	4
[33]	Yes	2	No	0	Naive and blind	2	No	0	4
[41]	Yes	2	Yes	2	Nothing	0	No	0	4
[30]	Yes	2	No	0	Blind to sham laser	1	No	0	3
[19]	Yes	2	No	0	Blind	1	No	0	3
[36]	No	0	Yes	2	Nothing	0	No	0	2
[23]	No	0	No	0	Nothing	0	No	0	0

Articles scoring ≤ 4/8 pts.

Of the various quality items that we scrutinized, all studies fulfilled the criteria on the validity of the outcome variable, all had measured the pain before and after the intervention, all had provided some estimate of the results in tables, graphs or text, and all had tested their results for statistical significance. The ANOVA was used for factorial designs in all studies in order to test how variables interacted or combined.

Other checklist items were not always fulfilled. Of the four quality criteria that we considered particularly important, namely unbiased/blind/naïve study subjects (item 1), random allocation (item 3), blinded assessment (item 9), and losses and exclusions (item 11), the first two were commonly satisfied (in 15 and 17 papers, respectively), whereas using unbiased/blind/naïve study subjects and accounting for losses and exclusions were ignored in many studies (present in 7 and 5, respectively). Five of the reviewed articles ( [18,23,36,43,44]) should be considered with care since their assessments of outcomes were not blinded.

Seven articles did not mention if the allocation of study subjects to each group was randomized. As it was not our intention to penalize unfairly articles that used a randomized allocation without mentioning it, we identified the numbers of individuals in each group in these reports (see Table 3). Articles [19,32,33] did not provide the number of subjects in each experimental group. The others (except [30]) showed a more even distribution than one could expect with a proper randomization procedure. We therefore assumed that the fact that authors did not discuss the randomization procedure, in most cases, reflected the fact that it had not occurred.

**Table 3:** Distribution of subjects in study groups in articles where allocation procedure was not specified

**Article**	**Total of subjects**	**SMT groups**	**Control group**	**No treatment**
[23]	60	30	30	30
[39]	30	10	10	10
[19]	10	?	?	?
[31]	15	7	8	-
[33]	18	?	?	?
[30]	50	19	18	13
[32]	24	?	?	?

In our review, we did not set a threshold for acceptable/unacceptable quality but displayed the articles in descending order from the highest to the lowest score. Nevertheless, in Table 2a, we compared the spread of articles when using the total score (18 point scale, see Table 2) and the scale based on four high-priority items (8 point scale, see Table 2b). With levels of “acceptance” provisionally set at 12/18 and 4/8, respectively, all articles, with one exception, obtained the same classification of acceptable or not, regardless of the system used. We interpreted this concordance as a confirmation of the robustness of the quality and risk of bias assessment.

In addition to the quality issues identified in this review, the authors themselves have in some instances discussed the limitations of their own studies (see Table 4).

**Table 4:** Limitations to own studies given by authors of articles reviewed for the effect of SMT on pain

	**Limitations given by authors**
[37]	Short term effect. Unable to project results on duration.
	Small sample of patients.
	Did not include control group.
[42]	4 different muscle situations assessed (rest, isotonic contraction and 2 isometric contractions) (Is it enough?)
	Duration (only immediate effect assessed).
	Pop sound may have a placebo effect.
[23]	Healthy subjects.
	Unable to describe duration of effects.
[28]	Pain-free patients.
	Style, contacts or force used in the mobilization procedures.
[29]	Duration. Unable to project results on duration.
	Widespread to other areas?
	Subthreshold pain stimulation, what about real pain?
	Latent trigger points, subjects who may not be typical population.
	Control group did not receive an intervention; maybe pop sound has a placebo effect.
[44]	Lack of control and placebo groups.
	Short term effect. Unable to project results on duration.
	Did not take into account subject innate stiffness.
[38]	Short term effect. Unable to project results on duration.
	Placebo effect of cavitation.
	Only women.
[30]	Algometer was not very precise.
[43]	-
[18]	Assessment not blind.
	Chronic low back pain.
	Temporal summation as an indirect measure of central sensitization has been proven only in animals.
[19]	Short term effect. Unable to project results on duration.
	Possible placebo effect of cavitation.
	Small sample of patients.
[39]	Short term effect. Unable to project results on duration.
	Not patients.
	Possible placebo effect of cavitation.
	PPT
[20]	Short term effect. Unable to project results on duration.
	Placebo effect of cavitation.
	Healthy people, not patients.
[31]	Short term effect. Unable to project results on duration.
	Possible placebo effect of cavitation.
	Healthy people, not patients.
[36]	Short term effect. Unable to project results on duration.
	Possible placebo effect of cavitation.
	Healthy people, not patients.
	No control, no sham group.
[40]	-
[21]	-
[32]	-
[33]	-
[41]	-
[35]	-
[34]	-

A meta-analysis was not attempted because the heterogeneity of studies on pain provoking methods, units of measurements, areas of the body where experiments were performed, and local, regional of systemic assessments of the effects of SMT.

### Data synthesis: Answers to research questions

Herein, each research question is dealt with one at a time, and for each, we refer to a table summarizing the results in order of quality of the study. If one study contains several experiments, they have all been reported individually in the appropriate section of the review. Table 5 presents studies grouped by their results, showing in the upper panels those that presented a hypoalgesic action and, in the lower panels, those that did not. Papers are also separated by the research question, i.e. local, regional or systemic effect. Table 6 summarizes for each article the effect of SMT on pain, the site where SMT was performed and the location of the pain. This table also shows values reported, whether the effect was local, regional or systemic, whether the effects were ipsilateral or contralateral, whether an effect occurred above or below the site of manipulation, and the type of pain induced.

**Table 5:** Effect of SMT on experimentally induced pain by localization of pain reduction

	**Systemic effect**	**Regional effect**	**Local or same metamere**
SMT relieved pain	[23]* -Cervical SMT/hand- foot T°	[42] – C5-C6 SMT / deltoid PPT	[37]– C5-C6 SMT/ Elbow PPT
	[44]* -Lumbar Mob/hand PPT	[29] – Atlantooccipital SMT/masseter PPT	[44] – Lumbar mob/ L2 L5 (foot) PPT
	[43]* - Lumbar SMT/ deltoid PPT	[38] - Atlantooccipital SMT/sphenoid PPT	[19] - C5-C6 SMT/ Elbow PPT
	[18]* -Lumbar SMT/hand- foot PPT	[30] - Lumbar SMT/ 1^st^ segment below PPT	[20] – C3-C4 SMT/ Trapezius PPT 5’
	[18]* -Lumbar SMT/hand- foot T°	[43]* - Lumbar SMT/ L3 PPT	[20] – C3-C4 SMT/ Trapezius PPT 10’
	[36]* -Lumbar SMT/hand- foot T°	[39] – C7-T1 SMT/ C5-C6 PPT	[31] - C5-C6 SMT/ Elbow PPT
		[39] – C7-T1 SMT/ C5-C6 PPT	[21] - C5-C6 SMT/ Elbow PPT
		[40] – Areas of stroking allodynia thorax SMT/ forearm	[21] - C5-C6 SMT/ Elbow spontaneous pain
		[40] – Mechanical hyperalgesia thorax SMT/ forearm	[32]- C5-C6 SMT/ Elbow PPT
		[40] – Spontaneous pain thorax SMT/ forearm	[33] - C5-C6 SMT/ Elbow PPT
			[33] –C5-C6 SMT/ Elbow stretch test
			[41]- Thoracic SMT/ spinous process electricity
			[35]- Thoracic SMT/ Thoracic PPT
SMT **did not** relieve pain	[23]* - Cervical SMT/hand- foot 1^st^ Pain T°	[37] – T5-T8 SMT (sham SMT)/ Elbow PPT	[42] – C5-C6 SMT / Trapezius PPT
	[23]* - Cervical SMT/hand- foot PPT		[28]** – C5-C6 Mob(AP)/ C5-C6 PPT
	[18]* – Lumbar SMT/hand- foot 1^st^ Pain T°		[19] - C5-C6 SMT/ Elbow Cold
	[36]* –Lumbar SMT/hand- foot 1^st^ Pain T°		[19] - C5-C6 SMT/ Elbow Hot
			[20]*** C3-C4 SMT/ Trapezius PPT0’
			[32] - C5-C6 SMT/ Elbow Temp PPT
			[33] –C5-C6 SMT/ Elbow spontaneous pain
			[34]- C0-C1SMT/ C2 PPT

*Assessment not blinded.

**This was an anterior-posterior mobilization, not lateral as SMT.

***PPT measured just after SMT, but there was a hypoalgesic effect at 5’ and 10’.

T° = temperature.

PPT = pressure pain thresholds.

**Table 5a:** Effect of SMT on experimentally induced pain by localization of pain reduction in relation to the quality of studies

	**Systemic effect**	**Regional effect**	**Local or same metamere**
SMT relieved pain	[23]* -Cervical SMT/hand- foot T°	[42] – C5-C6 SMT / deltoid PPT	[37] – C5-C6 SMT/ Elbow PPT
	[44]* -Lumbar Mob/hand PPT	[29] – Atlantooccipital SMT/masseter PPT	[44] – Lumbar mob/ L2 L5 (foot) PPT
	[43]* - Lumbar SMT/ deltoid PPT	[38] - Atlantooccipital SMT/sphenoid PPT	K - C5-C6 SMT/ Elbow PPT
	[18]* -Lumbar SMT/hand- foot PPT	[30] - Lumbar SMT/ 1^st^ segment below PPT	[20] – C3-C4 SMT/ Trapezius PPT 5’
	[18]* -Lumbar SMT/hand- foot T°	[43]* - Lumbar SMT/ L3 PPT	[20] – C3-C4 SMT/ Trapezius PPT 10’
	[36]* -Lumbar SMT/hand- foot T°	[39] – C7-T1 SMT/ C5-C6 PPT	[31] - C5-C6 SMT/ Elbow PPT
		[39] – C7-T1 SMT/ C5-C6 PPT	[21] - C5-C6 SMT/ Elbow PPT
		[40]– Areas of stroking allodynia thorax SMT/ forearm	[21]- C5-C6 SMT/ Elbow spontaneous pain
		[40] – Mechanical hyperalgesia thorax SMT/ forearm	[32]- C5-C6 SMT/ Elbow PPT
		[40] – Spontaneous pain thorax SMT/ forearm	[33] - C5-C6 SMT/ Elbow PPT
			[33] –C5-C6 SMT/ Elbow stretch test
			[41]- Thoracic SMT/ spinous process electricity
			[35]- Thoracic SMT/ Thoracic PPT
SMT **did not** relieve pain	[23]* - Cervical SMT/hand- foot 1^st^ Pain T°	[37] – T5-T8 SMT (sham SMT)/ Elbow PPT	[42] – C5-C6 SMT / Trapezius PPT
	[23]* - Cervical SMT/hand- foot PPT		[28]** – C5-C6 Mob(AP)/ C5-C6 PPT
	[18]* – Lumbar SMT/hand- foot 1^st^ Pain T°		[19]- C5-C6 SMT/ Elbow Cold
	[36]* –Lumbar SMT/hand- foot 1^st^ Pain T°		[19] - C5-C6 SMT/ Elbow Hot
			[20]*** – C3-C4 SMT/ Trapezius PPT0’
			[32] - C5-C6 SMT/ Elbow Temp PPT
			[33] –C5-C6 SMT/ Elbow spontaneous pain
			[34]- C0-C1SMT/ C2 PPT

X- : Studies scoring ≤ 12 points on 18 point scale **and** ≤ 4 points on 8 point scale.

X- : Study scoring ≤ 4 points on 8 point scale and more than 12 points on 18 point scale.

X *: Studies where the assessor was not blinded.

** This was an anterior-posterior mobilization, not lateral as is usually the case in SMT.

*** Pressure pain thresholds measured just after spinal manipulation, but there was a hypoalgesic effect at 5’ and 10’.

T° = temperature.

PPT = pressure pain thresholds.

**Table 6:** Detailed findings from literature review on effect of SMT on pain

**Art**	**Effect of SMT on pain**	**Site of SMT/pain**	**Values**	**Local/regional/ systemic effect**	**Same/opposite side**	**Above/below**	**Pain form**
[37]	The application of a cervical SMT, but not thoracic SMT, resulted in immediate bilateral hypoalgesic effect in patients with lateral epicondylitis.	SMT C5-C6 and T5-T8 /PPT both epicondyles	**Changes PPT in KiloPascals (differences)** PPT Cervical Thoracic affected side 88.6 (35.1%) 18.6 (0.8%) unaffected side 95.6 (25.4%) -40.5 (−0.9%)	Effect within the same segment. Used SMT caudal level as placebo with no effect.	No significant differences between L and R side	Effect within the same segment. Used SMT caudal level as placebo with no effect.	Pressure
[42]	On deltoid, small effect at the same segment. Didn't work on trapezius and C5	SMT C5-C6 right / PPT upper trapezius, deltoid and C5 spinous process	**Changes PPT in Kg/cmÂ² (differences)** PPT SMT Control Trapezius ipsilateral 0.2 0.3 Trapezius contralateral 0.4 0.1 Deltoid ipsilateral 0.3 -0.2 Deltoid contralateral 0.2 -0.2 C5 spinous process 0.1 -0.1	Small effect within the same segment	Comparison of sides baseline in Table 2, but no differences p>.523 Bilateral increases of PPT	-	Pressure
[23]	SMT reduced TSS (temporal sensory summation) but not PPT	Lower cervical and upper thoracic region / T° on hand + popliteal fossa	PPT increased for all groups (not only SMT) from pre to post SMT (F=9.6, partial NÂ²=0.10)= SMT produced a significant reduction in TSS (p=.003)	Averages of lower extremity values were higher than upper extremity values	-	SMT worked at the same level or below	Pressure and Temperature (T°)
[28]	No effect	Cervical mobilization left C5-C6 / PPT left and right articular pillar of C5-C6	**Differences PPT pre/post treatment** Kpa F=0.168 p=0.168 Treatment 15.98 (+/− 4.8%) Manual contact 4.61 (+/− 0.2%) No contact 12.29 (+/− 3.5%)	No effect at same segment	-	-	Pressure
[29]	Small immediate increase of PPT	SMT atlantooccipital/ PPT on trigger points in the masseter and temporalis muscles	Differences before/after SMT in Kg/cmÂ²: SMT = 0.29 (10%) Soft mobilization = 0.00 control= 0.019	Regional effect of atlantooccipital SMT and effect on trigeminal area	-	-	Pressure
[44]	Hypoaglesia significant at test site and without differences between the rates of mobilization	Lumbar mobilization/L2 dermatome(thigh), L5 (foot), hand and L5 paraspinal	Mean of changes: 19,6% paraspinal muscles 14,2% L2 dermatome 13,4% L5 dermatom 12% hand (this suggest that changes are systemic)	lumbar hypoalgesia was greater than distal (P=0.0028)	-	SMT more effective on lumbar dermatomes than more cephalad dermatomes	Pressure
[38]	Small effect regionally	SMT atlantooccipital/ PPT over both sides of sphenoid bone (V)	PPT effect on group and time F=14.4 (p<0.001) SMT = 3.5 kg/cmÂ² control = − 0.1 kg/cmÂ²	Regional level.	-	-	Pressure
[30]	Mobilization had a stronger effect on pain than SMT	SMT 1 segment below marked PPT (lumbar)	Mobilization = small increase (0.434 kg/cmÂ² d= 0.78) SMT = decrease ( −0.173 d= 0.36) Control = small decrease (−0.105 d= 0.25) but ANOVA further revealed non signification between groups.	Local and systemic effect but PPT values increase in a caudal direction	-	-	Pressure
[43]	No differences between amplitudes (p= 0.864)	lumbar mobilization/ 1- right erector spinae (L3) 2- left patella (L3 dermatome) 3- proximal lateral sruface of left 5th metatarsal (S1 dermatome) 4- deltoid	PPT A B C 1 18.73% 14.57% 15.48% 2 17.93% 9.93% 10.67% 3 10.53% 15.57% 8.81% 4 19.06% 18.60% 11.69%	Local and systemic effect	-	-	Pressure
[18]	Significant changes in temporal summation, only for SMT	Lumbar SMT/ temporal summation on plantar surface (non dominant) and palmar surface (non dominant). Aδ fibers mediated pain sensitivity in non dominant forearm and calf	**Lumbar (local response)**: A- Aδ fibers sensibility: no differences between groups at 47°C(p= .73), or 49°C (p= . 96) No effect of time at 47°C (p= .31) or 49°C (p= . 94) No changes in Aδ fibers B- temporal summation: F= 3,41 (p= . 05), different by group assignment = Changes in temporal summation **Cervical (general response)**: A- No changes in Aδ fibers B- temporal summation: SMT group F= 6,78 (p= . 40), all groups had a decrease in temporal summation = Changes in temporal summation	Systemic effect, except for first pain.	-	-	T°differences Numeric Rating Scale (0–100) Before/after: Bike = −3,7 LE Exercise = 2,5 SMT= 19,9
[19]	Effect demonstrated for PPT but not for T°	SMT C5-C6 dominant side (right) / PPT , thermal pain thresholds (HPT - CPT) on lateral epicondyles (both sides)	**Differences SMT Control** PPT ipsilateral 121.5 (44.2%) 13.3 ( 4.4%) PPT contralateral 74.4 (17.7%) 6.1(1.7%) HPT ipsilateral (°C) 1.2 (2.9%) 0.7 (2.2%) HPT contralateral 1.5 (4.1%) -0.9 (1.9%) CPT ipsilateral −0.25(9.2%) -1.5 (9.6%) CPT contralateral 0.9 ( 18.1%)-1.0 (17.4%)	Same segment	Bilateral increase of PPT. No significant changes for T°	-	Pressure and T°
[39]	SMT changes PPT in both R and L C5-C6 zygapophyseal joints in healthy subjects	SMT C7-T1 / PPT C5-C6 zygapophyseal joints	Differences on PPT before/after: **Right side** SMT dominant: 53.1 SMT non-dominant: 80.7 Placebo: -2.7 **Left side** SMT dominant: 45.9 SMT non-dominant: 48.0 Placebo: -3.9	Effect at regional level	SMT changes PPT in both R and L C5-C6 zygapophyseal. joints in healthy subjects	SMT is also effective above and below of segment treated	Pressure
[20]	SMT changes pressure pain sensitivity in triggers points in the upper trapezius	SMT C3-C4 / PPT upper trapezius trigger points (TrPs)	**Differences pre/post SMT in Kg/cmÂ² D**ifference Placebo SMT Pre post −0.06 d=0.35 0.08 d=0.4 Pre - 5' -0.2 d=1.1 0.1 d=0.5 Pre - 10' -0.22 d=1.1 0.12 d=0.44	Regional level	-	-	Pressure
[31]	SMT changes pressure pain sensitivity in epicondyles	SMT C5-C6 both sides / PPT on lateral epicondyles (both sides)	**Differences in Kg/cmÂ²** SMT ipsilateral 0.8 ( 35.5%) SMT contralateral 0.5 (24.8%) Placebo Ipsilateral 0.003(0.5%) Placebo contralateral 0.006 (0.4%) Control ipsilateral 0.003(0.5%) Control contralateral 0.006 (2.1%)	Same segment	No differences between L and R sides	-	Pressure
[36]	SMT produces hypoalgesia in lumbar area but not in cervical (control) but no effect on 1st pain	Lumbar SMT/ TSS in plantar surface (non dominant) and palmar surface (non dominant). Aδ fibers mediated pain sensitivity in non dominant forearm and calf	Lumbar Innervated NRS Change 47°C 13.2 (17.2) 12.9 (17.9) 23.5 (17.3) NRS Change 49°C 1.2 (20.2) 6.3 (22.4) 12.1 (19.7) Cervical Innervated NRS Change 47°C −3.0 (13.7) 0.3 (11.6) 0.3 (10.2) NRS Change 49°C 1.9 (9.0) -0.4 (10.1) 1.7 (10.8) NRS= Numeric rating scale	Effect at regional level but not at systemic level	-	Effect at the same level, but not above	T°
[40]	Allodynia and hyperalgesia decrease with SMT	Non specific thoracic SMT / left and right forearm (capsaicin)	Pre-SMT Post-SMT Pre-sham Post-Sham Hyperalgesia(cmÂ²) 53 31 39 56 Allodynia (cmÂ²) 40 18 28 40 Spontaneous pain (ratings) 4.9 3.3 3.9 4.2	SMT decreases allodynia at regional level	-	-	Capsaicin
[21]	Effect on PPT	SMT C5-C6 / PPT over symptomatic segment, T°PT	PPT increases p: < 0.05 +/− 0.? % control +/− 2.?% placebo +/− 22.55 SMT VAS didn't work	Regional level	-	-	Pressure
[32]	Mobilization has an effect on pressure pain, not on temperature	Mobilization C5-C6 / PPT both elbows	PPT increases p: < 0.05 +/− −4.? % control +/− −7.?% placebo +/− 29 % SMT TPT didn't work	Regional level.	-	-	Pressure and T°
[33]	Increase of PPT	SMT cervical C5-C6 / PPT both elbows	**Changes pre/post treatment** SMT +/− 26% Placebo +/− −12% Control +/− 0.2%	Regional level.	-	-	Pressure
[41]	Elevation of pain tolerance in manipulated group	Thoracic manipulation/ Electric thresholds left and right articular pillar	**Intensity of current in mAmp** SMT Control Baseline 1.37 1.62 30'' 2.05 1.46 2 min 2.43 1.46 5 min 2.70 1.56 10 min 3.30 1.86	Local level.	-	-	Electrical induction
[34]	No significant differences	SMT C0-C1/ PPT C2	preHVLA-HVLA -5’ -39.37 (76.07) Kpa (SD) preHVLA-HVLA-30’ -15.89 (87.50) preMET-MET-5’ -42.03 (62.37) preMET-MET-30’ -30.00 (69.53) preControl-Control-5’ -15.88 (83.62) preControl-Control-30’ -16.12 (62.49)	Local level.	-	-	Pressure
[35]	Mobilization and manipulation both produced a statistically significant increase in PPT in the thoracic spines of asymptomatic subjects. Mobilization more than SMT.	Thoracic manipulation T1-T4/ PPT on most tender thoracic vertebra	**Differences PPT pre/post treatment** Kpa (SD) Manipulation Mobilisation Control Pre-intervention 243.70 (95.22) 204.6 (85.52) 218.71 (82.91) Post-intervention 244.64 (91.59) 216.51 (90.50) 47.13 (96.87) Difference 0.94 (35.07) 11.88 (31.83) 28.42 (39.68)	Local level.	-	-	Pressure

T° = temperature.

PPT = pressure pain thresholds.

HPT = hot pain threshold.

CPT = cold pain threshold.

In the majority of experiments (28/43), SMT reduced pain. Outcomes were not dependent on whether quality scores were high or low. These studies demonstrate a clear hypoalgesic effect of SMT (see Table 5a). For specific results, see below.

1. Does SMT reduce experimentally induced pain provoked by pressure? There were 27 experiments performed on pressure pain. Nineteen of these showed manipulation to increase pressure pain thresholds (PPT). There was no obvious link between the reported polarity of the effect and the quality of the studies. Changes produced by SMT were in general statistically significant. Differences in PPT values (before/after SMT), when reported as percentages ranged between 4.8% (in [28]) and 44.2% (in [19]). Other units of measurement were also used making it difficult to summarize with a single parameter the size of the effect. For detailed information, see Tables 6 and Additional file 3.

2. Does SMT reduce experimentally induced pain provoked by temperature? There were 9 experiments on pain induced by temperature, only 3 of which showed a hypoalgesic action for SMT: studies [18,23,36] found that SMT relieves pain provoked by temperature and therefore transmitted by C-fibers. The other experiments (n = 6) did not show significant differences in relation to SMT. Three of these tested first pain (A fiber system) and the other three tested TSS (C-fiber system). There was no obvious link with the quality of studies. For values and detailed information, see Table 6 and Additional file 4.

3. Does SMT reduce experimentally induced pain provoked by methods other than pressure and temperature? Only 7 tests were performed using methods other than pressure and temperature to induce pain. Six of these revealed a statistically significant hypoalgesic effect induced by SMT. There did not seem to be a link with the quality of studies. For values and detailed information, see Tables 6 and Additional file 5.

4. Does SMT reduce experimentally induced pain in the spinal segment where it is performed? Twenty experiments investigated whether SMT reduces experimentally induced pain in the spinal segment where it is performed. Twelve of them showed a hypoalgesic effect. The other eight presented no significant effects. Of these, study [28] tested the hypoalgesic action of an anterior-posterior mobilization and not a lateral maneuver, as is usually the case in SMT. In study [20], there was no effect immediately after SMT, but an effect was demonstrable five and ten minutes later. In studies [19,31] differences between sides were studied, but no difference was found. Outcomes did not relate to the quality score. For detailed information, see Tables 5 and 6.

5. Does SMT reduce experimentally induced pain in the same region where it is performed? Nine experiments reported a regional effect of SMT on pain. Only one study failed to obtain a hypoalgesic effect. In this, thoracic manipulation was used as a sham treatment versus cervical manipulation to evaluate the action on elbow PPT. There did not seem to be a link between treatment effects reported and the quality of studies. For detailed information, see Table 5 and 6.

6. Does SMT have a systemic effect on experimentally induced pain? Nine experiments evaluated this hypothesis. None of them had blinded assessors, which makes the results uncertain. Five of them demonstrated a systemic action of SMT on pain, but four of them did not show significant differences between treatment groups. Three of these four (studies [18,23,36]) evaluated first pain transmitted by A fibers. There did not seem to be a link between treatment effects reported and the quality of studies. For detailed information, see Tables 5 and 6.

### Additional observations

1. No article presented any data on the duration of the pain reduction. Studies [43,44] concluded that the hypoalgesic effect was unrelated to the amplitude of the manual procedure, whereas [30] concluded that mobilization had a stronger effect than manipulation.

2. Four studies ( [29,35,36,40]) applied the SMT to a point on the spine thought to be in need of treatment, i.e. at what was considered to be a dysfunctional spinal segment (manipulable lesion/fixation/subluxation), whereas in all other studies the exact location of SMT was predetermined without reference to local signs or symptoms. However, hypoalgesic results were observed regardless of whether the treatment was provided in a “clinical” fashion, i.e. where the patient would have what the clinician considered a dysfunctional segment, or if the manipulation was given in a predetermined area.

3. In two studies ( [20,29]) the investigators searched for so-called trigger points.

4. Five articles ( [19,31,37,39,42]) assessed whether the effect of SMT was only on the ipsilateral side of the impulse or if it was also noted on the contra-lateral side. None of the trials found a side-specific effect.

5. Several reports (N = 9) made reference to the “crack” ( [18-20,23,29,37-39,42]), which is thought to occur when a joint is cavitated [14]. If no cavitation was obtained, a second manipulation would be given. There was no obvious difference in results between studies that concentrated on the crack and those that did not. However, none studied that issue specifically, making it impossible to know whether all study subjects were “cracked” or not.

6. Regarding whether the effect could be noticed above or below the segment where the SMT was performed, results lacked consistency. In two of the studies ( [23,39]), an effect was noted below the area of treatment. Studies [39,44] showed an effect above the area of treatment, whereas two of the studies ( [36,37]) did not detect an effect above or below the relevant segment.

7. Outcomes were not affected by whether the control group received a sham treatment or no treatment at all.

8. In 10 of the experiments (5 articles), patients with current pain were included ( [18,19,21,32,33]). Three of these experiments ( [19,21,32]) found increased PPT values after SMT. Article [33] showed relief of pain on stretching painful muscles after SMT but not relief of spontaneous pain, whereas article [21] reported that spontaneous pain was diminished by SMT. Articles [18,19] evaluated temperature-induced pain with similar results, that is to say there was a hypoalgesic action on second pain [18], no action on first pain [18] and no action in 2 trials on TSS.

## Discussion

### Summary of results

At the time of conducting this review, only one earlier systematic review was available on this subject. It examined 11 articles but was unable to draw conclusions regarding treatment effects [45]. For the present review, however, it was possible to identify 22 relevant articles, which made it possible to draw several conclusions in relation to the possible pain-reducing effect of SMT. Thus our results indicate clearly that such an effect is achievable.

However, differences in effect exist according to whether the outcome is tested locally, regionally or systemically. An effect was clearly shown locally and regionally, whereas an effect is less clear in more distant parts of the body. Also the outcome differs according to the method of pain induction; pain induced by pressure, electricity, stretching of painful tissue, dermal irritation, and spontaneous pain all respond to SMT, whereas temperature-induced pain does not always respond.

### Methodological considerations of our own review

One limitation of this review is that there is no generally accepted and validated quality check list for the type of experimental studies which we examined and so we had to select our own quality criteria, based on some basic methodological concepts important to our research questions. Such a check list can be modified, which could affect the overall quality assessment. According to our quality scoring system, studies scored between 8 and 16 points out of a possible 18. However, in order to discriminate better between studies, a more detailed scale could have resulted in different scores, which could perhaps have separated studies into more obviously “good” and “bad”. However, the relevance, if any, of these quality items in relation to outcome, is not known and, in fact, the quality scores were not clearly associated with outcomes.

The strengths of this review are that the search for relevant articles was free of language bias, that the constituent articles were reviewed independently by two reviewers, that the results can be considered in relation to the quality of studies, that there was no arbitrary threshold for acceptable quality [46], that the check list tables are sufficiently detailed to allow readers to perform their own analysis of the information provided, and that there was a relatively large number of studies, making it possible to examine several research questions.

### Methodological considerations for the studies under review

This review identified some common methodological problems, such as lack of blinded assessment that could weaken the evidence in these experimental studies. We considered one of the most important points to be that the assessor was blinded, to avoid expectation bias. Some studies ignored this issue. This aspect affected our interpretation of the systemic effect of SMT, as all five articles (10 trials) dealing with the systemic effects of SMT lacked a blinded assessor.

It is also important that the results be truthfully presented, and not exaggerated in some way, meaning that any subjects or data excluded from analysis should be accounted for. This was often ignored.

Another challenge was that of the control group. Ideally, the SMT should be matched against a suitable sham treatment and control procedure, and this was done in 12 studies. This is difficult with physical procedures, but several of the other 10 studies tried to make the best of the situation by selecting naive study subjects, and in some cases different types of treatment were compared (such as treatment in two areas of the spine), in which case no sham treatment would be necessary. However, whether a proper sham treatment was used or not, there were no obvious differences in the results, possibly indicating that the effect is very obvious and not affected to a large extent by expectations.

Potential confounders of these effects would be anxiety in general and fear of pain, known moderators of treatment outcomes in clinical practice. However, according to the three articles in which anxiety and fear of pain were studied, there were no statistically significant correlations between pain-related cognition, pain thresholds and the hypoalgesic response to SMT, indicating that the psychological aspect, considered to be so important in the perception of pain, perhaps does not come into play during experimental studies of this type.

### Comparison with another systematic review

Some of our results were corroborated by a newly published high quality review on this very topic [47]. This other review included 15 of our articles but, as they did not put a restriction on duration of symptoms in symptomatic people, they also incorporated 5 articles not included in our review. They defined effects as occurring “locally” or in “remote” areas, and pooled the results in all studies for the effects of PPT. They found the effects on PPT to be small in both areas but nevertheless statistically significant. When the data were separated for local and remote effect significance was only noted for the remote effect. They did not study any other pain–inducing method nor did they take into consideration the quality of studies, although they did perform a quality assessment.

### Discussion of findings

Pain provoked by pressure, electricity, stretching of painful tissue, dermal irritation, or spontaneous pain all respond to SMT.

Experimental pain provoked by pressure was the most common method used to assess the effect of manipulation (27 out of 43 experiments). Unfortunately, the reports did not use the same units for reporting increased tolerance to pressure, making it difficult to compare outcomes, but SMT was shown to increase PPT values between 4.8% and 44.6%. That the algometer, used for this purpose, is a highly reliable method to measure pain [48] suggests that these results are robust. However, they cannot necessary be compared between studies because of different experimental situations.

Nevertheless, it is not clear whether these changes are clinically significant. One author [44] referred to Moss [49], who stated that a 15% improvement in pressure pain tolerance is clinically important, but this statement rests on previous studies which dealt with osteoarthritic pain [50] and pain in an emergency service [51], not experimental pain. Therefore, the cut-off point at which the reduction of artificially induced pain has reached a clinically significant level is not known.

In addition, it cannot be concluded that the effect of SMT, as seen in these studies, would be as large in "naturally" painful areas. Therefore, it is not certain that the degree of pain reduction detected in experimental studies can be extrapolated to other situations.

Apart from pressure and temperature, other sources of pain were: capsaicin to irritate the skin followed by skin stroking to evoke allodynia, hyperalgesia induced by mechanical means, and spontaneous pain. In these instances also, SMT was generally able to reduce pain.

Pain provoked by temperature does not always respond to SMT.

Nine trials from five papers examined the effects of SMT on temperature induced pain. In 3 of the 6 tests examining second pain, an effect was found but none of the 3 trials evaluating first pain could demonstrate such an effect. This indicates that SMT may have an effect on C-fiber mediated “second pain” but not on the more acute “first pain” mediated by A-delta fibers. This finding may help to resolve the mechanisms by which SMT reduces pain.

How broadly does the effect extend?

Almost all authors discussed the possibility of a systemic effect of SMT but this was tested in only 5 studies (9 experiments). The results were clearly positive, although two of the authors concluded that local effects were stronger than more distant ones. However, none of these studies used a blinded assessor, and so the reported results must be treated with caution.

Nine experiments out of the ten that studied the regional effect were positive (see Table 5). However, in the reviewed articles, it was difficult to differentiate between strictly local vs. more regional effects. The definition of regional is uncertain if following the dermatomes, as they are known to differ from the text-book mappings [52] and the origins and distributions of cutaneous nerves differ from person to person. Thus, a negative outcome in one person may simply be due to that person having an unusual pattern of nerve distribution. Despite this, most studies showed a positive effect with relatively few study subjects. This could indicate either that negative results were removed from the analyses or that the effect is consistent.

Imprecise dermatomal mapping makes it difficult not only to separate clearly local from regional effects but also regional from systemic effects. Interestingly, the application of spinal manipulation at the atlanto-occipital region was found to have an effect on the masseter muscle, despite that muscle being supplied by the trigeminal nerve, which does not exit in the upper cervical spine. We therefore described this experiment under “regional” rather than “local” but it could perhaps equally well have been described it as “systemic”.

Concerning the action of SMT on pain produced above or below the manipulated segment, only a few studies dealt with this and no consistent findings emerged. It would be necessary to test this issue specifically, in order to work out whether manipulation-induced impulses travel up or down the spinal cord or whether both occur.

Five studies investigated whether the hypoalgesic effect was mainly on the side of the manipulation or if it also appeared on the opposite side. These studies consistently demonstrated a bilateral effect.

Some clinical concepts

Although this review was based on experimentally induced pain, four findings emerged that could have a bearing on clinical practice, or at least on the concepts on which clinical practice is based. Manipulation of a “restricted motion segment”, sometimes referred to as a “manipulable lesion”, was not required for the “treatment” to have an effect. Additionally, the side of the manipulation was irrelevant. However, the results from this review can only be considered from a clinical point of view, if SMT has a similar effect on pain in a clinical context, which was not investigated in this study.

Finally, although a hypoalgesic effect was shown, it is not known how long this effect lasts; long enough for a person with pain to be able to regain a normal movement pattern or only long enough to give an impression of improvement? Other effects of the SMT were not studied in this review; effects such as improved biomechanics or reduced inflammation of disturbed tissues [53].

### Implications in relation to research

There is a need to establish a coordinated global research strategy on the subject of SMT and pain relief. It seems unnecessary to conduct further research on simply whether SMT has an effect on pain or not. Rather, future work should focus on more precise questions such as why SMT does not affect first pain, and which mechanisms are involved in relieving pain locally, regionally and systemically. The magnitude and duration of effects also need to be defined. Consideration needs to be given to the most appropriate research designs for addressing different questions.

There could also be more consistency in outcome measures, and closer attention should be paid to important design elements such as blinded assessment, random allocation, appropriate control groups and, if possible, the recruitment of naïve subjects.

### Implications in relation to education and clinical practice

Some of the findings in this review do not support the imperative of specificity, i.e. precise identification of a manipulable lesion and the exact level and side of the manipulation.

## Conclusions

This systematic critical review of the literature confirms an effect of spinal manipulative therapy (SMT) on experimentally induced pain in human beings. This hypoalgesic effect seems to be local/regional and more consistent for pain provoked by pressure than by temperature. Further and better research is needed to determine if there is a systemic effect, to determine the exact mechanisms by which SMT relieves pain, the clinical importance and duration of the hypoalgesic effect.

## Competing interests

Authors declare there are no conflicts of interest.

## Authors contributions

All authors instigated this review. MM and CLY designed the check-lists, reviewed the literature and wrote the first draft. BB and MAA provided expertise on the topic, assisted with the literature review and provided critical comments to the first draft. All authors reviewed the final manuscript and approved the final version.

## Supplementary Material

Additional file 1 **Inclusion and exclusion criteria in a survey of the effect of spinal manipulative therapy on experimentally induced pain [**[2,46,54-60]. Click here for file

Additional file 2 The items selected for the quality checklist and their rationale. Click here for file

Additional file 3 Effects of SMT on pressure pain thresholds (PPT). Click here for file

Additional file 4 Effects of SMT on pain produced by temperature. Click here for file

Additional file 5 Effects of SMT on pain produced by methods other than pressure or temperature. Click here for file
