# Supplementary material for: The effect of spinal manipulative therapy on experimentally induced pain: a systematic literature review
Source: Chiropr Man Therap. 2012 Aug 10;20:26. doi: 10.1186/2045-709X-20-26 (PMC3527169; doi:10.1186/2045-709X-20-26)
Supplement: Additional file 1 — Inclusion and exclusion criteria in a survey of the effect of spinal manipulative therapy on experimentally induced pain [[2,46,54-60]. [file 2045-709X-20-26-S1.doc]

**Additional file 1:** Inclusion and exclusion criteria in a survey of the effect of spinal manipulative therapy on experimentally induced pain

- Year: no restrictions.
- Languages: no restrictions.
- Pain, anywhere, experimental. In order to identify the primary effect of SMT on pain, it would be preferable to study experimentally induced pain in relatively healthy people i.e. to avoid chronic syndromes that may have a secondary effect on pain and attitudes towards pain. These inclusion criteria eliminate confounding of the hypoalgesic response by clinical conditions and pain medications, and the experience of pain is well controlled in contrast to spontaneously experienced pain. In addition, healthy volunteers can easily communicate quality and intensity of painful stimuli, and human pain models appear to be ideally suited to test analgesic therapies . That is why there are a number of human experimental pain models, such as the cold pressor model , the intramuscular injection of hypertonic saline solution , the heat/capsaicin sensitization and intradermal capsaicin models or pressure pain thresholds .
- Spinal manipulative therapy (SMT), anywhere in the spine.
- Humans or animals: no restrictions.
- Immediate effect, i.e. we were not interested in the application of SMT one day with effects measured perhaps weeks or months later.
- Experimental studies with at least one external or internal control group.

Studies were excluded if they presented one of the following characteristics:

- Chronic pain, defined as pain of more than one year. Chronic pain has its own mechanisms of modulation which would complicate the experimental situation.
- Pathological conditions (e.g. osteoarthritis, disc herniation, neurogenic pain, and neuropathy) that could confuse the experimentally induced pain with already existing symptoms .
- Combined/ concomitant therapies (drugs, physiotherapy), as it would not be possible to know if the results were obtained from SMT or the other therapies.
- Studies about only spinal motion or tenderness, because the aim of this review is to clarify the effect of SMT on pain.
- Reviews.
- Case reports.
