# Supplementary material for: The effect of spinal manipulative therapy on experimentally induced pain: a systematic literature review
Source: Chiropr Man Therap. 2012 Aug 10;20:26. doi: 10.1186/2045-709X-20-26 (PMC3527169; doi:10.1186/2045-709X-20-26)
Supplement: Additional file 2 — The items selected for the quality checklist and their rationale. [file 2045-709X-20-26-S2.doc]

**Additional file 2:** The items selected for the quality checklist and their rationale.

In relation to study subjects

1. Were study subjects unbiased? Study subjects could be influenced by their expectations and for this reason it was important that they were either blind to the nature of the experiment, or that they were at least naïve to the topic and that they did not have a strong interest in the outcome of the experiment.
2. Was there control of psychological characteristics of subjects that could affect pain perception? The reason for this is that, for example, fear of pain or expectations could modify the perception of pain.
3. Was allocation to study group randomized? We did not deal with the appropriateness of the method to randomly allocating subjects into one study group or the other but were satisfied if, at least, such a procedure was mentioned.

In relation to the experiment

1. Was the intervention (SMT) well described with sufficient details to allow replication?
2. Was the intervention (SMT) performed by the same person for all experiments and was this person competent/experienced?
3. Was the number of trials (experimental pain and manipulation) stated? If the number of trials was stated we assumed that it would be less likely that some results would have been removed from the analyses.
4. If trials were not conducted on the same day, were the before and after measurements undertaken under near-identical circumstances, such as in the same room, at the same temperature? It also seems to be important to repeat the experiment at the same time of the day to eliminate changes produced by circadian variations.
5. Had the outcome variable(s) previously been shown to be reproducible and valid? Or was this tested in the study? Or was there at least a pilot study to ensure optimal study circumstances?

In relation to the assessment

1. Was the assessor blinded to group allocation? This item was considered very important because the assessor could be subconsciously influenced by the wish to obtain “good” results.
2. Was pain measured both before and after the SMT?

In relation to analysis and data reporting

1. Were losses and exclusions reported?
2. Were estimates of the results given or shown in a graph? This would make it possible to determine treatment effect size.
3. Were differences between study groups tested for statistical significance?
