# Supplementary material for: The effect of spinal manipulative therapy on experimentally induced pain: a systematic literature review
Source: Chiropr Man Therap. 2012 Aug 10;20:26. doi: 10.1186/2045-709X-20-26 (PMC3527169; doi:10.1186/2045-709X-20-26)
Supplement: Additional file 3 — Effects of SMT on pressure pain thresholds (PPT). [file 2045-709X-20-26-S3.doc]

## Additional file 3 - Effects of SMT on pressure pain thresholds (PPT).

|  | **Interventions** | **Site of pain** | **Results of SMT on pressure pain thresholds** | **Significant Effect: yes/no** | **Quality score** |
| --- | --- | --- | --- | --- | --- |
|  | SMT C5-C6 Sham procedure Nothing* | PPT over symptomatic segment | Effect on PPT: SMT more effective than control SMT more effective than placebo | Yes | 15 |
|  | SMT C5-C6 right Nothing | PPT upper trapezius ipsilateral PPT upper trapezius contralateral | No significant differences | No | 14 |
|  | SMT C5-C6 right Nothing | PPT deltoid ipsilateral PPT deltoid contralateral | SMT increases PPT (no differences for sides) | Yes | 14 |
|  | SMT C5-C6 right Nothing | C5 spinous process | No significant differences | No | 14 |
|  | SMT atlantooccipital  Soft tissue Nothing | PPT on trigger points in the masseter and temporalis muscles | SMT increases PPT | Yes | 14 |
|  | Lumbar mobilization 2 Hz Lumbar mobilization 1 Hz Lumbar mobilization Quasi stable | L2 dermatome (thigh), L5 (foot) | Hypoalgesia significant at test site and  without differences between the rates of mobilization (but local>distal) | Yes | 14 |
|  | Lumbar mobilization 2 Hz Lumbar mobilization 1 Hz Lumbar mobilization Quasi stable | Hand and L5 paraspinal | Hypoalgesia significant of site test and  without differences between the rates of mobilization (but local>distal) | Yes | 14 |
|  | SMT atlantooccipital Sham procedure | PPT over both sides of sphenoid bone (V) | SMT increases PPT | Yes | 14 |
|  | Manipulative thrust right side C7-T1 Manipulative thrust left side C7-T1 Sham-manual procedure | PPT on left dominant C5-C6 zygapophyseal joints PPT on right dominant C5-C6 zygapophyseal joints | SMT changes PPT in both R and L C5-C6 zygapophyseal joints in healthy subjects Right side more than left side | Yes | 14 |
|  | Manipulative thrust right side C7-T1 Manipulative thrust left side C7-T1 Sham-manual procedure | PPT on left non-dominant C5-C6 zygapophyseal joints PPT on right non- dominant C5-C6 zygapophyseal joints | SMT increases PPT in both R and L C5-C6 zygapophyseal joints in healthy subjects Right side greater than left side | Yes | 14 |
|  | SMT atlantooccipital  Muscle energy technique Nothing | PPT next to C1 | No significant differences | No | 14 |
|  | Thoracic manipulation T1-T4  Thoracic mobilization  Control sham laser | PPT on most tender thoracic vertebra | Mobilization has a stronger effect on pain than SMT | Yes | 14 |
|  | SMT C5-C6 | PPT on lateral epicondyles (both sides) | SMT increases PPT | Yes | 13 |
|  | SMT T5-T8 (sham SMT) | PPT on lateral epicondyles (both sides) | No significant differences | No | 13 |
|  | Cervical mobilization left C5-C6 (2Hz) (A-P) Sham procedure Nothing | PPT left and right articular pillar of C5-C6 | No significant differences | No | 13 |
|  | Lumbar mobilization - large oscillation (force applied) Lumbar mobilization small oscillation Lumbar mobilization quasi static | 1- Right erector spinae (L3)  2- Left patella (L3 dermatome) | Hypoalgesia significant at site of test. No differences between amplitudes | Yes | 13 |
|  | lumbar mobilization - large oscillation (force applied) lumbar mobilization small oscillation lumbar mobilization quasi static | 3- Proximal lateral surface of left 5th metatarsal (S1 dermatome)  4- Deltoid | Hypoaglesia significant at site of test. No differences between amplitudes. | Yes | 13 |
|  | SMT C3-C4 Sham procedure | PPT upper trapezius trigger points (TrPs) immediate control | No significant differences | No | 13 |
|  | SMT C3-C4 Sham procedure | PPT upper trapezius trigger points (TrPs) 5 minute control | SMT changes pressure pain sensivity in MTrPs in the upper trapezius | Yes | 13 |
|  | SMT C3-C4 Sham procedure | PPT upper trapezius trigger points (TrPs) 10 minute control | SMT changes pressure pain sensivity in MTrPs in the upper trapezius | Yes | 13 |
|  | SMT C5-C6 both sides Sham procedure Nothing | PPT on lateral epicondyles (both sides) | SMT changes pressure pain sensivity in epicondyles No differences between sides. | Yes | 13 |
|  | SMT C5-C6 dominant side (right) Sham procedure | PPTon lateral epicondyles (both sides) | SMT increases PPT. Right side greater than left side. | Yes | 11 |
|  | SMT cervical C5-C6 Sham procedure Nothing | PPT both elbows | Increase of PPT | Yes | 11 |
|  | Unilateral HVLA Spinal lumbar mobilization Sham laser procedure | SMT 1 segment below marked PPT (lumbar) | Mobilization has a stronger effect on pain than SMT | Yes | 10 |
|  | SMT cervical C5-C6 Sham procedure Nothing | PPT both elbows | Increase of PPT | Yes | 9 |
|  | SMT Lower cervical and upper thoracic region Cervical exercises Nothing | Hand | No significant differences | No | 8 |
|  | SMT Lower cervical and upper thoracic region Cervical exercises Nothing | Popliteal fossa | No significant differences | No | 8 |

*Nothing means that the study subject did not receive any type of procedure.
