# Supplementary material for: The effect of spinal manipulative therapy on experimentally induced pain: a systematic literature review
Source: Chiropr Man Therap. 2012 Aug 10;20:26. doi: 10.1186/2045-709X-20-26 (PMC3527169; doi:10.1186/2045-709X-20-26)
Supplement: Additional file 4 — Effects of SMT on pain produced by temperature. [file 2045-709X-20-26-S4.doc]

## Additional file 4 - Effects of SMT on pain produced by temperature.

|  | **Interventions** | **Site of pain** | **Effects of SMT on temperature-induced pain** | **Significant Effect: Yes/no** | **Quality score** |
| --- | --- | --- | --- | --- | --- |
|  | Lumbar SMT Back extension exercise Biking | Plantar surface (non dominant) and palmar surface (non dominant). Temporal summation. | Significant changes in temporal summation only for SMT | Yes | 12 |
|  | Lumbar SMT Back extension exercise Biking | Non dominant forearm and calf. Aδ fiber-mediated pain sensitivity. | No differences concerning first pain (Aδ fibers) | No | 12 |
|  | SMT C5-C6 dominant side (right) Sham procedure | Lateral epicondyles (both sides). Heat pain threshold. | No significant differences | No | 11 |
|  | SMT C5-C6 dominant side (right) Sham procedure | Lateral epicondyles (both sides). Cold pain threshold. | No significant differences | No | 11 |
|  | SMT cervical C5-C6 Sham procedure Nothing | TPT both elbows | No significant differences | No | 11 |
|  | Lumbar SMT Bicycle riding Lumbar extension exercise | Plantar surface (non dominant) and palmar surface (non dominant).  47°^C^ / 49°^C^ - Temporal summation. | SMT produces hypoalgesia in lumbar area but not in cervical (control) | Yes | 10 |
|  | Lumbar SMT Bicycle riding Lumbar extension exercise | Non dominant forearm and calf. 47°^C^ / 49°^C^ - Aδ fiber-mediated pain sensitivity. | No significant differences on first pain for both | No | 10 |
|  | SMT Lower cervical and upper thoracic region Cervical exercises Nothing | Hand Popliteal fossa | SMT reduces temporal sensory summation | Yes | 8 |
|  | SMT Lower cervical and upper thoracic region Cervical exercises Nothing | Hand Popliteal fossa | No differences concerning first pain (Aδ fibers) | No | 8 |
