# Supplementary material for: The effect of spinal manipulative therapy on experimentally induced pain: a systematic literature review
Source: Chiropr Man Therap. 2012 Aug 10;20:26. doi: 10.1186/2045-709X-20-26 (PMC3527169; doi:10.1186/2045-709X-20-26)
Supplement: Additional file 5 — Effects of SMT on pain produced by methods other than pressure or temperature. [file 2045-709X-20-26-S5.doc]

## Additional file 5 - Effects of SMT on pain produced by methods other than pressure or temperature.

|  | **Interventions** | **Site of pain** | **Results** | **Significant Effects: Yes/no** | **Quality score** |
| --- | --- | --- | --- | --- | --- |
|  | Mobilization 3° degree C5-C6  Sham mobilization  Nothing | VAS, spontaneous pain | SMT vs. control : bigger differences  SMT vs. placebo: smaller differences | Yes | 15 |
|  | Thoracic SMT  Sham procedure | Areas of stroking allodynia on forearm | Allodynia decreases with SMT | Yes | 13 |
|  | Thoracic SMT  Sham procedure | Mechanical hyperalgesia on forearm | SMT more effective - Left/right not reported | Yes | 13 |
|  | Thoracic SMT  Sham procedure | Spontaneous pain intensity on forearm | SMT more effective - Left/right not reported | Yes | 13 |
|  | Thoracic SMT  Sham procedure | Electrical induction 1" lateral to spinous process of T1/T10 | SMT more effective - Left/right not reported | Yes | 10 |
|  | SMT cervical C5-C6  Sham procedure  Nothing | VAS, spontaneous pain on elbows | No significant differences | No | 9 |
|  | SMT cervical C5-C6  Sham procedure  Nothing | Elbows. Stretch test. | SMT more effective | Yes | 9 |
